# Effects of ALDH2 Genotype, PPI Treatment and L-Cysteine on Carcinogenic Acetaldehyde in Gastric Juice and Saliva after Intragastric Alcohol Administration

**DOI:** 10.1371/journal.pone.0120397

**Published:** 2015-04-01

**Authors:** Ryuhei Maejima, Katsunori Iijima, Pertti Kaihovaara, Waku Hatta, Tomoyuki Koike, Akira Imatani, Tooru Shimosegawa, Mikko Salaspuro

**Affiliations:** 1 Division of Gastroenterology, Tohoku University Graduate School of Medicine, Sendai, Japan; 2 Research Unit on Acetaldehyde and Cancer, University of Helsinki, Helsinki, Finland; Shiga University of Medical science, JAPAN

## Abstract

Acetaldehyde (ACH) associated with alcoholic beverages is Group 1 carcinogen to humans (IARC/WHO). Aldehyde dehydrogenase (ALDH2), a major ACH eliminating enzyme, is genetically deficient in 30–50% of Eastern Asians. In alcohol drinkers, ALDH2-deficiency is a well-known risk factor for upper aerodigestive tract cancers, i.e., head and neck cancer and esophageal cancer. However, there is only a limited evidence for stomach cancer. In this study we demonstrated for the first time that ALDH2 deficiency results in markedly increased exposure of the gastric mucosa to acetaldehyde after intragastric administration of alcohol. Our finding provides concrete evidence for a causal relationship between acetaldehyde and gastric carcinogenesis. A plausible explanation is the gastric first pass metabolism of ethanol. The gastric mucosa expresses alcohol dehydrogenase (ADH) enzymes catalyzing the oxidation of ethanol to acetaldehyde, especially at the high ethanol concentrations prevailing in the stomach after the consumption of alcoholic beverages. The gastric mucosa also possesses the acetaldehyde-eliminating ALDH2 enzyme. Due to decreased mucosal ALDH2 activity, the elimination of ethanol-derived acetaldehyde is decreased, which results in its accumulation in the gastric juice. We also demonstrate that ALDH2 deficiency, proton pump inhibitor (PPI) treatment, and L-cysteine cause independent changes in gastric juice and salivary acetaldehyde levels, indicating that intragastric acetaldehyde is locally regulated by gastric mucosal ADH and ALDH2 enzymes, and by oral microbes colonizing an achlorhydric stomach. Markedly elevated acetaldehyde levels were also found at low intragastric ethanol concentrations corresponding to the ethanol levels of many foodstuffs, beverages, and dairy products produced by fermentation. A capsule that slowly releases L-cysteine effectively eliminated acetaldehyde from the gastric juice of PPI-treated ALDH2-active and ALDH2-deficient subjects. These results provide entirely novel perspectives for the prevention of gastric cancer, especially in established risk groups.

## Introduction

Acetaldehyde associated with the consumption of alcoholic beverages is classified by the International Agency for Research on Cancer (IARC) as a Group 1 human carcinogen [1]. A causal relationship has been identified between acetaldehyde and esophageal cancer. This is based on epidemiological and biochemical findings on alcohol drinking ALDH2-deficient individuals [1,2]. Due to a point mutation in the gene encoding aldehyde dehydrogenase (ALDH2), its activity is undetectable in homozygotes and far less than half of the normal level in heterozygotes [3]. Over 500 million people with Eastern Asian roots have been estimated to be carriers of the deficient enzyme [4]. If they are heavy drinkers, their risk of oral, pharyngeal, and esophageal cancers is over 10-fold higher than in drinkers with the normal ALDH2 enzyme [5]. Consistently with epidemiological findings, alcohol drinking in ALDH2-deficient subjects results in markedly elevated concentrations of acetaldehyde in saliva [6–8]. Thus, ALDH2-deficient alcohol drinkers provide a unique human model for increased local exposure of the upper aerodigestive tract mucosa via saliva to carcinogenic acetaldehyde [6–9].

In individuals with the active ALDH2 enzyme, the capacity of the liver to eliminate acetaldehyde formed from ethanol is so efficient that measurable levels of acetaldehyde are not detected in the peripheral blood [10]. The highest concentrations of acetaldehyde after alcohol consumption are found in saliva, which is in the vicinity of the target organ sites known to be susceptible to ethanol-induced cancer [6,8]. Oral microbes and mucosal cells possess alcohol dehydrogenase (ADH) and are responsible for the majority of acetaldehyde produced in saliva in the presence of ethanol [7,11,12]. Acetaldehyde accumulates into the oral cavity and the saliva, since oral mucosal cells and microbes have very low levels of aldehyde dehydrogenase and thus a limited capacity to catabolize acetaldehyde [11–13].

Alcohol is also a significant risk factor for gastric cancer [14]. The highest gastric cancer risk has been found among ALDH2-deficient heavy drinkers with atrophic gastritis [15]. Atrophic gastritis is a major risk factor for gastric cancer and also increases the risk of esophageal squamous cell carcinoma, especially in ALDH2-deficient subjects [16–18]. An acid-free stomach secondary to atrophic gastritis or proton pump inhibitor (PPI) treatment is colonized with oral microbes, which leads to the enhanced local production of acetaldehyde from ethanol after alcohol administration [19,20].

L-cysteine, a semi-essential amino acid, is able to eliminate acetaldehyde by covalently binding to it. The product is the stable and inactive 2-methylthiazolidine-4-carboxylic acid [21,22]. Preparations that slowly release L-cysteine have been successfully used to eliminate acetaldehyde from saliva during alcohol consumption and smoking and from gastric juice after alcohol ingestion [23–25].

The effect of ALDH2 deficiency on the acetaldehyde concentration in gastric juice after alcohol administration is not yet known. Neither is the effect of a slowly L-cysteine releasing capsule on gastric juice and salivary acetaldehyde levels of PPI-treated ALDH2-active or -deficient subjects in the presence of ethanol. Therefore, the aim of this study was to determine the effects of the ALDH2 genotype, PPI treatment and a slowly L-cysteine-releasing capsule on the acetaldehyde levels in gastric juice and saliva after intragastric alcohol administration.

## Materials and Methods

### Study design and subjects

10 ALDH2-active (ALDH2-1/2-1) and 10 ALDH2-deficient (ALDH2-1/2-2) *H. pylori*-negative healthy volunteers were enrolled in the study, which was conducted at Tohoku University Hospital in Japan. All subjects were non-smokers and normal social drinkers. Healthy Japanese males aged over 20 years old were eligible for inclusion. All subjects were confirmed to be *H. pylori*-negative by a urea breath test before enrolment. Subjects were excluded if they had a habit of smoking or consuming large amounts of alcohol (over 60 g of alcohol daily), could not drink any alcohol, were taking any potent gastric acid suppressive drugs, had experienced gastrointestinal tract resection, or had ongoing therapy for any serious disease.

All participants completed the following aspiration study before and after the 7-day administration of a proton pump inhibitor (PPI, rabeprazole 10 mg b.i.d: after breakfast and after dinner) (study 1 & study 2). After 3 more days on PPI, another study was repeated with the administration of 200 mg of slowly L-cysteine releasing capsules (Acetium, Biohit Oyj., Helsinki, Finland) immediately before the ethanol infusion (study 3). The study was conducted in accordance with the Helsinki Declaration, and was approved by the Ethics Committee for Human Research at Tohoku University School of Medicine, Sendai, Japan (2012-2-107-1). All subjects provided written informed consent prior to enrollment in the study. The study was registered at the University Hospital Medical Information (UMIN000012378).

### Aspiration study procedure

The volunteers were asked to refrain from consuming alcohol for 24 hours and food for 12 hours prior to each study, and they came to our unit for each study between 1 and 3 PM.

A nasogastric tube (10 Fr, 91 cm; Salem Sump tube; Covidien, Dublin, Ireland) was inserted into the subjects to a depth of 55 cm after local anesthesia of the nasal cavity with lidocaine jelly (Xylocaine 2% jelly, AstraZeneca, Sweden) containing no ethanol. The position of the tube in the stomach was confirmed by aspiration of gastric juice. During the study, the volunteers were asked to lie on their left side to delay gastric emptying and thus enable the collection of gastric juice samples. Gastric aspirates and saliva were collected for subsequent analysis (Time 0). Then, ethanol (0.5 g/kg body weight) diluted in water to 15% w/vol solution was infused via the nasogastric tube into the stomach. This amount of alcohol corresponds to about a standard dose of alcohol. Thereafter, 5 ml of gastric aspirates and 1 ml of saliva were collected at 30-min intervals for up to 120 min (time 30, 60, 90, and 120 min). For the Acetium study (study 3), each volunteer was asked to swallow two Acetium capsules (Biohit Oyj., Helsinki, Finland; total 200 mg) with a minimal amount of water immediately before the ethanol infusion. When gastric juice could not be aspirated, 10 ml of distilled water was infused via the nasogastric tube and gastric juice was then collected. The samples were analyzed for the concentration of acetaldehyde and ethanol. The pH of the gastric juice before ethanol infusion was measured for each study using a pH meter (HORIBA D-51 model pH meter, HORIBA Co. Ltd., Kyoto, Japan). The laboratory work was performed so that the investigators were blinded to the subjects’ medical information.

### Acetaldehyde and ethanol analysis of gastric juice and saliva samples

To measure the acetaldehyde concentration, samples comprising 3 sets of 450 μl gastric juice and 1 set of 450 μl of saliva were transferred into a headspace vial containing 50 μl of 6 mol/l perchloric acid to stop microbial acetaldehyde production. In our unpublished tests, perchloric acid has been shown not to hydrolyze cysteine-acetaldehyde binding [25]. The samples were immediately deep frozen and stored at −80°C until transfer on dry ice to Biomedicum Helsinki, Finland, for further analysis. Acetaldehyde and ethanol levels of the gastric juice and saliva samples were analyzed by headspace gas chromatography, as previously described [6,11]. Two parallel samples were used for measurements and the mean value was calculated.

### *H. pylori* status and ALDH2 polymorphism genotyping

The *H. pylori* infection status was determined by a ^13^C urea breath test before enrollment using an infrared spectrophotometer (POCone^©^, Otsuka Electronics, Co, Osaka, Japan). The genomic DNA of each subject was extracted from EDTA anticoagulated peripheral blood leukocytes using a QIAamp DNA Blood Mini Kit (Qiagen, Valencia, CA). Genotyping for the *ALDH2* Glu504Lys polymorphism (rs671) was determined by TaqMan SNP genotyping assays on an Applied Biosystems StepOne real-time PCR system (Foster City, CA, USA). The subjects were divided into two groups: those homozygous for active alleles (*ALDH2-1/2-1*), referred to as ALDH2-actives, and those heterozygous with an inactive allele (*ALDH2-1/2-2*), referred to as ALDH2-deficients.

### Slowly L-cysteine releasing capsules (Acetium)

The capsules (Acetium, Biohit Oyj., Helsinki, Finland) contained 100 mg of L-cysteine as an active ingredient. L-cysteine is bound with matrix granules including Eudragit RS-PO, Hypromellose (HPMC), calcium hydrogen phosphate (CaHPO4) and titanium dioxide. The formulation causes L-cysteine to be released at a sustained rate and locally in the stomach.

### Statistical analysis

Clinical parameters of the volunteer profiles are presented as means ± SEM for continuous variables. Gastric juice and salivary acetaldehyde concentrations and areas under the curve (AUCs) of acetaldehyde in gastric juice and saliva were calculated and are expressed as means ± SEM. Statistical differences between the ALDH2-active and ALDH2-deficient groups were analyzed using the unpaired Student’s t-test. This was applied because in some samplings a small amount of water had to be infused to the stomach in order to facilitate proper sampling of gastric juice. We considered a *P* value of less than 0.05 to be statistically significant. All statistical analyses were conducted using Graph Pad Prism version 6.03 (Graph Pad Inc, San Diego, CA, USA) or t JMP 10 (SAS Institute Inc., Cary, NC, USA).

## Results

### Participant profiles

One ALDH2-deficient subject experienced nasal and stomach pain after alcohol infusion in study 1, and subsequent studies consequently had to be canceled in this subject. The other 19 subjects completed the entire study protocol and experienced no adverse events during the study period. There were no significant differences in background characteristics of ALDH2-active and ALDH2-deficient groups regarding their age and body size (mean age 26.9 ± 1.9 and 24.8 ± 2.3 years; mean body weight 62.8 ± 1.6 and 67.8 ± 2.7 kg in ALDH2-actives and -deficients, respectively).

### pH of fasting gastric juice

The pH of fasting gastric juice before rabeprazole administration (study 1) was comparable between ALDH2-active and ALDH2-deficient groups (2.6 ± 0.5 vs. 2.3 ± 0.5). One week of rabeprazole administration profoundly elevated the fasting intragastric pH to near neutral levels in study 2, and the pH was maintained at a similar level in study 3, irrespective of the *ALDH2* genotype (ALDH2-active vs. ALDH2-deficient groups: 6.9 ± 0.2 vs. 6.6 ± 0.4 in study 2 and 6.9 ± 0.2 vs. 6.6 ± 0.7 in study 3, respectively). There were no significant differences in gastric juice pH values between the two *ALDH2* genotypes in any studies.

### Gastric juice and salivary acetaldehyde levels after intragastric infusion of alcohol

#### Effect of ALDH2 genotype (study 1)

**Fig 1:**
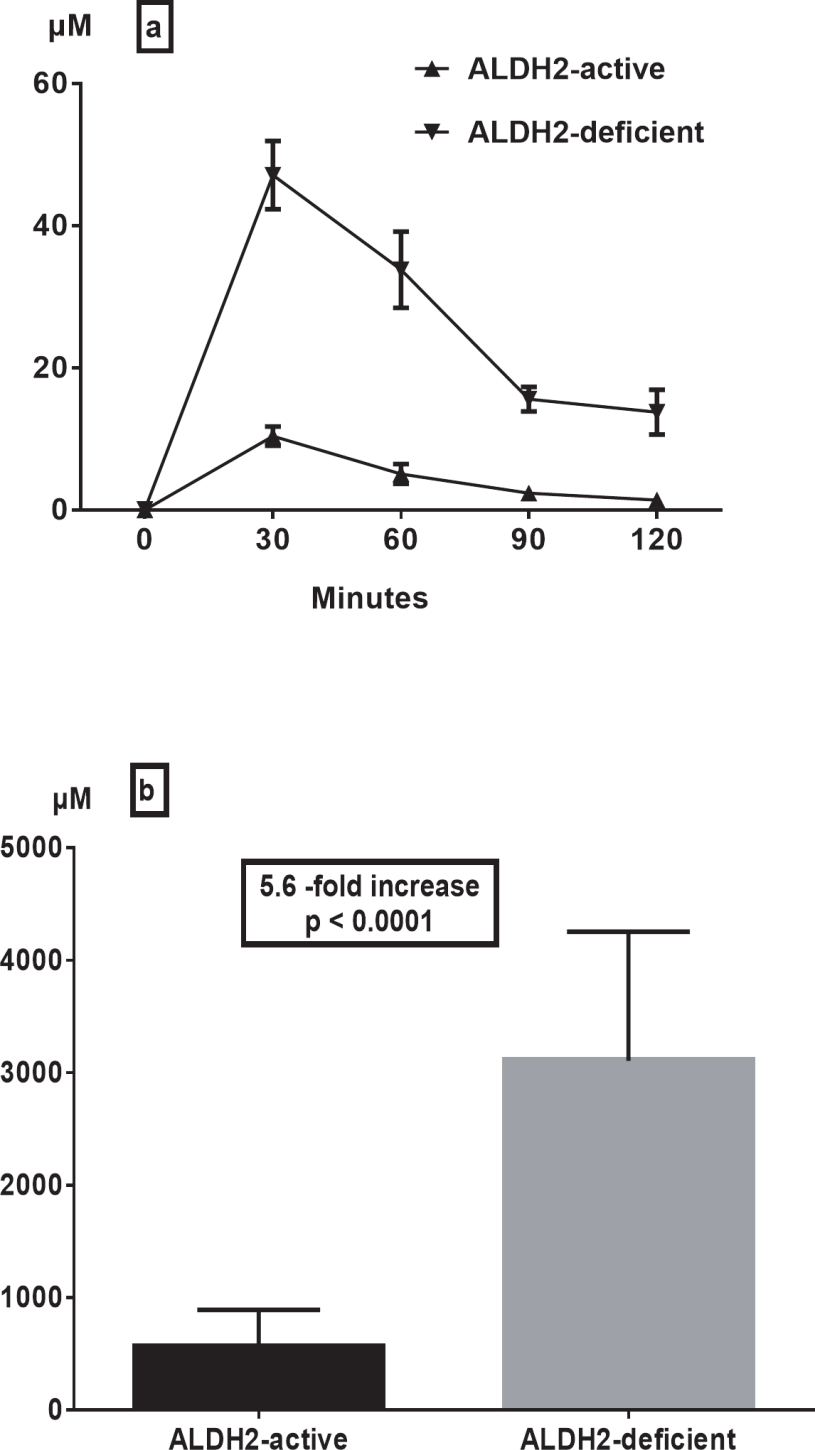
Effect of ALDH2 deficiency on gastric juice acetaldehyde levels (a) and areas under the curve (b) after intragastric infusion of ethanol (15 vol%, 0.5 g/kg) (means ± SEM).

Intragastric alcohol infusion yielded diverse effects on the acetaldehyde concentration of the gastric juice depending on the *ALDH2* genotype. While the intragastric alcohol infusion caused only a slight increase (mean peak at 30 min 10.4 ± 1.4 μM, range 2.3–17.4 μM) in the gastric juice acetaldehyde concentration of ALDH2-active subjects, a profound elevation in this concentration was seen in ALDH2-deficient subjects (mean peak at 30 min 47.1 ± 4.8 μM, range 31.3–70.5 μM) (Fig. 1A). Furthermore, gastric juice acetaldehyde levels of ALDH2-deficients remained constantly higher than those of ALDH2-actives throughout the study period of 120 minutes (Fig. 1A). Quantitatively, intragastric alcohol infusion resulted in a mean increase of 5.6-fold in the area under the curve (AUC) of gastric juice acetaldehyde in ALDH2-deficient subjects compared to that of ALDH2-active subjects (Fig. 1B, p < 0.0001).

**Fig 2:**
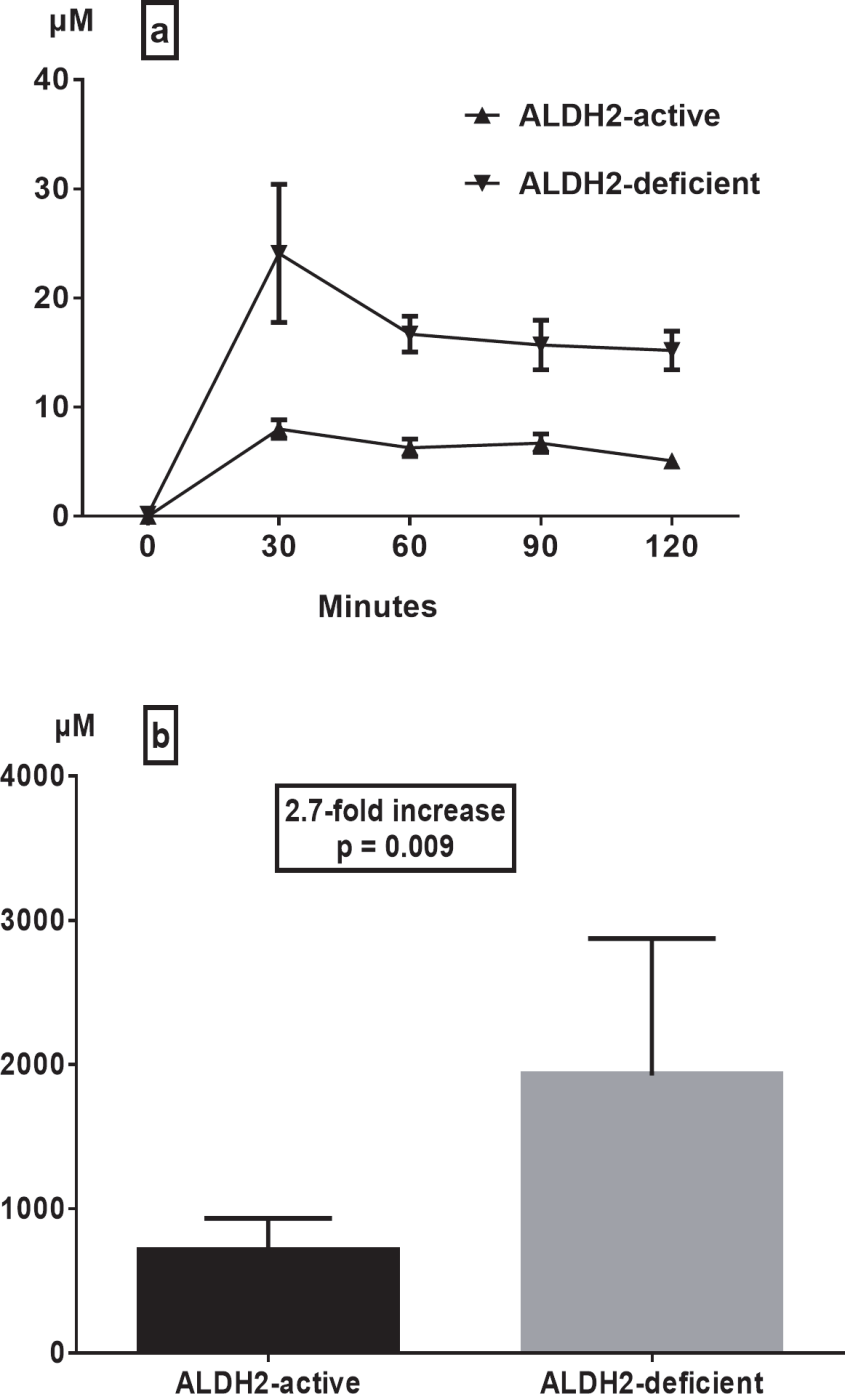
Effect of ALDH2 deficiency on salivary acetaldehyde levels (a) and areas under the curve (b) after intragastric infusion of ethanol (15 vol%, 0.5 g/kg) (means ± SEM).

Salivary acetaldehyde concentrations after intragastric alcohol infusion were also higher in ALDH2-deficient subjects (mean peak at 30 min 24.1 ± 6.3 μM, range 9–79 μM) as compared to those of ALDH2-active subjects (mean peak at 30 min 8 ± 0.8 μM, range 5–12 μM) (Fig. 2A). Consistently with gastric juice acetaldehyde, salivary acetaldehyde levels of ALDH2-deficients also remained constantly higher than those of ALDH2-actives throughout the study period of 120 minutes. Thus, the mean AUC of salivary acetaldehyde in ALDH2-deficient subjects was 2.7 times higher than that of ALDH2-active subjects (Fig. 2B, p = 0.009).

#### Effect of PPI treatment (study 2)

**Fig 3:**
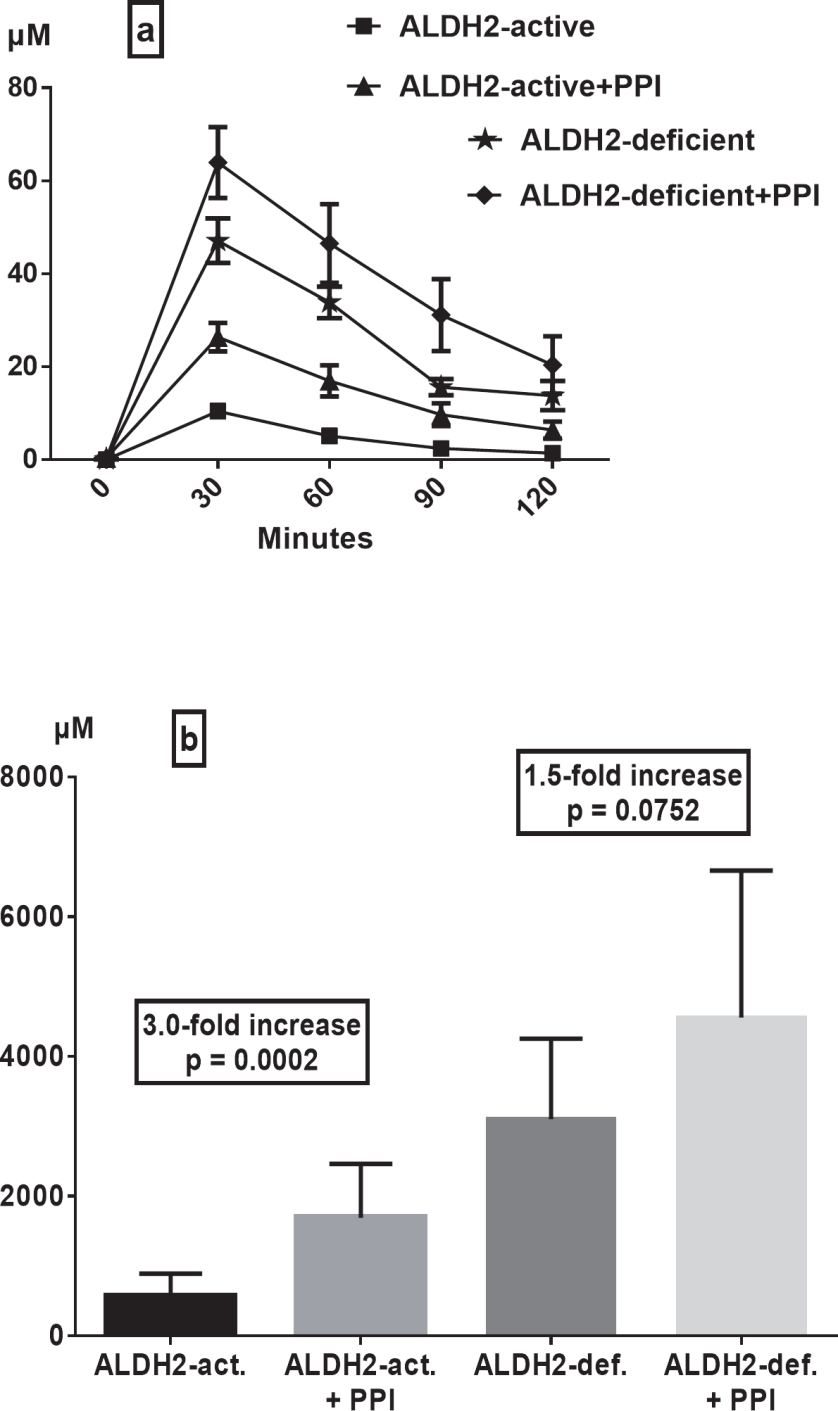
Effect of PPI treatment (rabeprazole 10 mg b.i.d. for 7 days) on gastric juice acetaldehyde levels (a) and areas under the curve (b) after intragastric infusion of ethanol (15 vol%, 0.5 g/kg) in ALDH2-active and -deficient subjects (means ± SEM).

In ALDH2-active subjects, the administration of rabeprazole (10 mg b.i.d.) for 7 days markedly increased the gastric juice acetaldehyde concentration, with a mean peak concentration at 30 min of 26.4 ± 3.1 μM (range 9.3–43.0 μM) (Fig. 3A). Consequently, in ALDH2-actives, the mean AUC of gastric juice acetaldehyde was 3.0-fold higher than in the study without rabeprazole (Fig. 3B, p = 0.0002). Similarly, in ALDH2-deficient subjects, rabeprazole treatment caused a trend towards a further increase in the gastric juice acetaldehyde concentration, with the mean peak level at 30 min being 63.9 ± 7.7 μM (range 32.0–96.7 μM) (Fig. 3A). The last value was the highest gastric juice acetaldehyde concentration observed in any of the study conditions. The mean AUC of gastric juice acetaldehyde under rabeprazole treatment of ALDH2-deficients was 1.5-fold higher than that of ALDH2-actives (Fig. 3B, p = 0.075). However, due to large individual variation in gastric juice acetaldehyde concentrations, the increase was not statistically significant.

In contrast to gastric juice acetaldehyde, rabeprazole had no effect on the acetaldehyde concentration in saliva in either *ALDH2* genotype group (data not shown).

#### Effect of the capsule releasing L-cysteine

**Fig 4:**
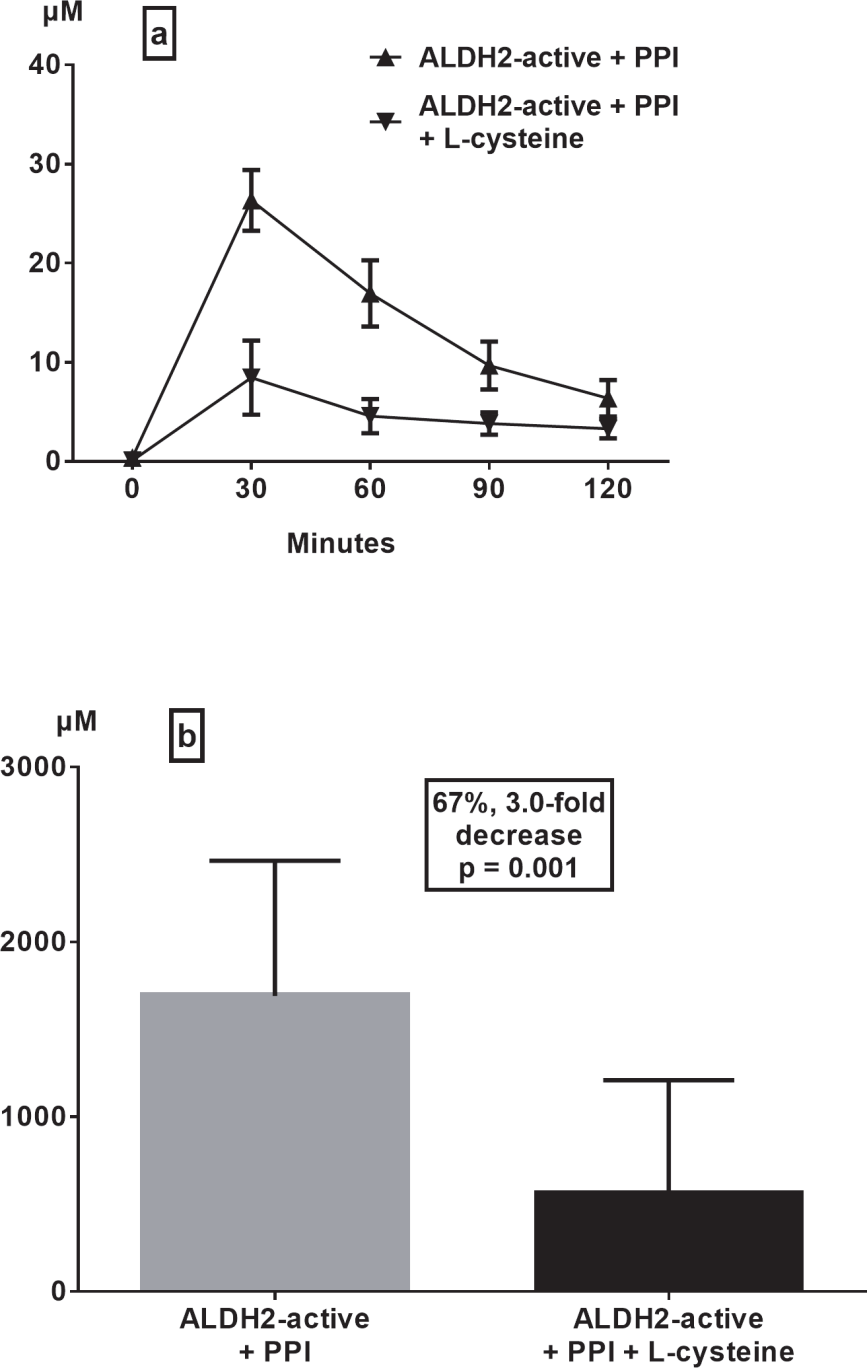
Effect of slowly L-cysteine releasing capsules (2 x 100mg) in PPI-treated ALDH2-active individuals on gastric juice acetaldehyde levels (a) and areas under the curve (b) after intragastric infusion of ethanol (15 vol%, 0.5 g/kg) (means ± SEM).

**Fig 5:**
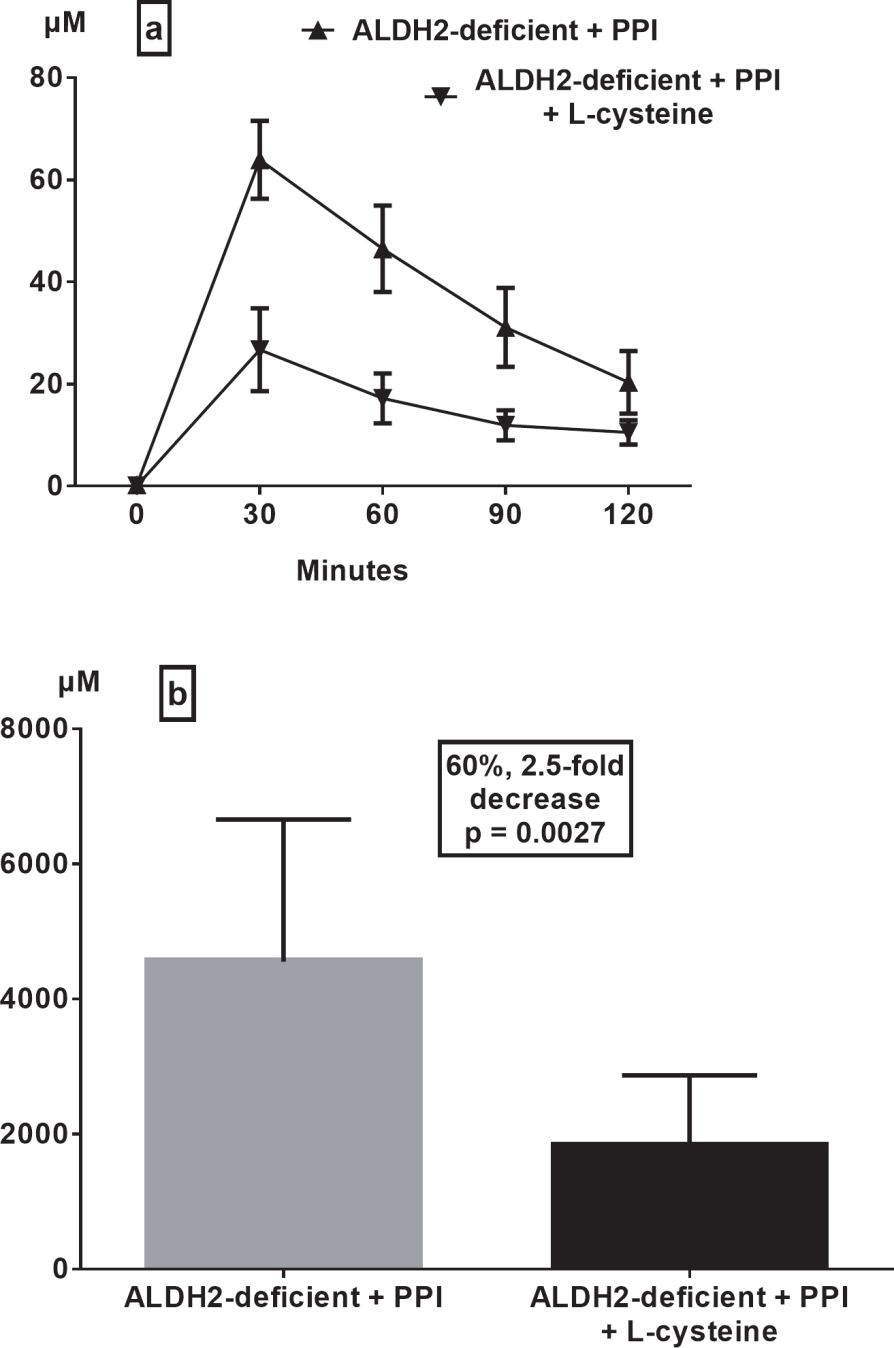
Effect of slowly L-cysteine releasing capsules (2 x 100) in PPI-treated ALDH2-deficient individuals on gastric juice acetaldehyde levels (a) and areas under the curve (b) after intragastric infusion of ethanol (15 vol%, 0.5 g/kg) (means ± SEM).

In ALDH2-active subjects, L-cysteine administration markedly reduced the gastric juice acetaldehyde concentration from the mean peak value of 26.4 ± 3.7 μM (range 9.3–43.0 μM) at 30 min without L-cysteine to a mean peak of 8.4 ± 3.7 μM (range 0.2–35.5 μM) at 30 min with L-cysteine (Fig. 4A). The acetaldehyde-eliminating effect of L-cysteine lasted almost the whole follow-up period of 120 minutes (Fig. 4A). Quantitatively, L-cysteine resulted in a mean decrease of 67% (3-fold) in gastric juice acetaldehyde (AUC) (Fig. 4B, p = 0.001). Similarly, L-cysteine greatly reduced the gastric juice acetaldehyde concentration in ALDH2-deficient subjects from a mean peak value of 63.9 ± 7.7μM (range 32.0–96.7 μM) at 30 min without L-cysteine to a mean peak of 26.7 ± 8.1 μM (range 3.8–51.2 μM) at 30 min with L-cysteine (Fig. 5A), and the effect again persisted throughout the follow-up period of 120 minutes (Fig. 5A). Consequently, L-cysteine resulted in a 60% (2.5-fold) reduction in the mean AUC of gastric juice acetaldehyde in ALDH2-deficient subjects (Fig. 5B, p = 0.0027).

L-cysteine had no effect on the salivary acetaldehyde concentration in either ALDH2 genotype group (data not shown).

#### Gastric juice and salivary ethanol concentrations

Gastric juice ethanol concentrations generally followed similar pattern in studies 1, 2 and 3 in both ALDH2-actives and ALDH2-deficients (Table 1). Mean peak gastric juice ethanol levels at 30 min ranged from 2.6 ± 0.6 vol% to 4.4 ± 0.9 vol% (from 572 ± 132 mM to 968 ± 198 mM). At 120 min, mean gastric juice ethanol levels ranged from 0.038 ± 0.001 vol% to 0.231 ± 0.131 vol% (from 8.4 ± 2.2 mM to 50.8 ± 28.8 mM). It should be noted that from the time points of 60 minutes to 120 minutes, gastric juice acetaldehyde levels were rather low, ranging from 1.22 to 0.038 vol%, i.e. from 12.2‰ to 0.38‰.

**Table 1:** Ethanol levels (vol %) in gastric juice and saliva after intragastric administration of ethanol (15 vol%, 0.5 g/kg) (means ± SEM).

Characteristics	30 minutes	60 minutes	90 minutes	120 minutes
**Gastric juice**				
***Study 1 (Before PPI)***				
ALDH2-actives	3.68 ± 0.50	0.82 ± 0.24	0.23 ± 0.05	0.091 ± 0.011
ALDH2-deficients	3.89 ± 0.59	0.98 ± 0.23	0.29 ± 0.11	0.128 ± 0.034
***Study 2 (+ PPI)***				
ALDH2-actives	3.74 ± 0.54	0.77 ± 0.15	0.23 ± 0.05	0.091 ± 0.016
ALDH2-deficients	4.03 ± 0.76	1.22 ± 0.36	0.40 ± 0.15	0.171 ± 0.059
***Study 3 (+ PPI + L-cys.)***				
ALDH2-actives	2.56 ± 0.57	0.54 ± 0.17	0.12 ± 0.02	0.067 ± 0.008
ALDH2-deficients	4.36 ± 0.87	1.15 ± 0.33	0.44 ± 0.19	0.231 ± 0.131
**Saliva**				
***Study 1 (Before PPI)***				
ALDH2-actives	0.074 ± 0.004	0.056 ± 0.002	0.059 ± 0.010	0.041 ± 0.004
ALDH2-deficients	0.096 ± 0.005	0.070 ± 0.003	0.055 ± 0.004	0.044 ± 0.002
***Study 2 (+ PPI)***				
ALDH2-actives	0.087 ± 0.006	0.057 ± 0.005	0.048 ± 0.002	0.041 ± 0.002
ALDH2-deficients	0.097 ± 0.008	0.070 ± 0.005	0.054 ± 0.004	0.042 ± 0.003
***Study 3 (+ PPI, + L-cys.)***				
ALDH2-actives	0.086 ± 0.005	0.057 ± 0.003	0.045 ± 0.002	0.038 ± 0.001
ALDH2-deficients	0.092 ± 0.007	0.073 ± 0.004	0.056 ± 0.004	0.046 ± 0.002

Ethanol concentrations in saliva also followed similar pattern in all studies in both ALDH2-actives and ALDH2-deficients (Table 1). Mean peak salivary ethanol concentrations at 30 min ranged from 0.074 ± 0.004 vol% to 0.097 ± 0.008 vol% (from 16.3 ± 0.9 mM to 21.3 ± 2.0 mM). At 120 min mean salivary acetaldehyde levels ranged from 0.038 ± 0.001 vol% to 0.046 ± 0.002 vol% (from 8.4 ± 0.2 mM to 10.1 ± 0.4 mM).

## Discussion

### Effects of ALDH2-deficiency

In the present study, we demonstrated for the first time that a genetic deficiency of the ALDH2 enzyme results in markedly increased exposure of the gastric mucosa to acetaldehyde after intragastric administration of alcohol. When combined with earlier epidemiological findings, our results provide concrete evidence for the casual relationship of acetaldehyde not only with the pathogenesis of esophageal squamous cell cancer, but also with gastric carcinogenesis [1,2,18,26,27]. A plausible explanation for the ethanol-induced elevation of gastric juice acetaldehyde is the gastric first pass metabolism of ethanol, i.e. the local oxidation of ethanol in the stomach [28]. The gastric mucosa expresses at least two types of alcohol dehydrogenase (ADH) that produce acetaldehyde from ethanol, as well as an acetaldehyde-eliminating ALDH2 enzyme [29]. The findings of this study indicate that due to deficient ALDH2, the gastric mucosa is not able to eliminate acetaldehyde produced from ethanol by gastric mucosal ADH enzymes, resulting in the accumulation of acetaldehyde in gastric juice.

Mutagenic DNA adducts increase exponentially at acetaldehyde concentrations from 25 to 500 μM [30]. These acetaldehyde concentrations were reached after intragastric alcohol administration in the gastric juice of both ALDH2-deficient and PPI-treated subjects. Peak gastric juice acetaldehyde levels were seen at 30 min after alcohol infusion, when ethanol concentrations in the gastric juice were highest. These findings are in line with the kinetic properties of gastric mucosal ADH enzymes, which metabolize ethanol faster at the high ethanol concentrations prevailing in the stomach shortly after the consumption of alcoholic beverages [28,29,31].

In earlier studies, it has been demonstrated that after alcohol consumption the salivary acetaldehyde concentrations of ALDH2-deficient subjects are 2–3 times higher than in those with the normal ALDH2 enzyme [6,8]. In the present study, it was shown that intragastric administration of alcohol also results in similarly elevated acetaldehyde concentrations in saliva. Thus, our results support earlier conclusions indicating that the increased levels of acetaldehyde found in the saliva of ALDH2-deficient individuals are derived from the parotid glands, which produce acetaldehyde from systemic ethanol but have a limited capacity to detoxify it [6,7,9].

### Effects of PPI treatment

Atrophic gastritis is a major risk factor for gastric cancer and also significantly increases the risk of esophageal squamous cell carcinoma, especially in ALDH2-deficient subjects [16–18]. A recent meta-analysis suggested that the use of acid-suppressive drugs is also associated with an increased risk of stomach cancer [32]. A common pathogenetic denominator in both cases is acetaldehyde endogenously formed from ethanol [33,34]. An achlorhydric stomach secondary to either atrophic gastritis or treatment with proton pump inhibitors is colonized by oral microbes, which effectively produce acetaldehyde from ingested alcohol via their ADH enzymes [19,20]. In accordance with these earlier findings, rabeprazole treatment for 7 days also highly significantly increased gastric juice acetaldehyde levels in ALDH2-active subjects after intragastric infusion of a moderate dose of alcohol, and the effect lasted for 120 minutes. Highest gastric juice acetaldehyde concentrations were measured in rabeprazole-treated ALDH2-deficient subjects.

### Effects of slow-release L-cysteine

Acetaldehyde is a cytotoxic, genotoxic and mutagenic compound and carcinogenic in experimental animals [1,2]. When associated with the consumption of alcoholic beverages, it has been classified as carcinogenic to humans (Group 1) [1]. The new classification concerns both the acetaldehyde present in alcoholic beverages as well as when endogenously formed from ethanol, e.g. by local microbial or mucosal oxidation of ethanol to acetaldehyde in the upper aerodigestive tract [1]. Due to its aldehyde group, acetaldehyde is very reactive and easily binds to various tissue components, including DNA, and forms carcinogenic DNA adducts [2]. A recent animal model study demonstrated increased formation of carcinogenic DNA adducts in the gastric mucosa of ALDH2 knockout mice treated with ethanol [35]. Acetaldehyde is also effectively and covalently bound to L-cysteine by forming a stable 2-methylthiazolidine carboxylic acid [21]. This binding results in the inactivation of the reactive aldehyde group. L-cysteine is a semi-essential amino acid that is widely used as a food additive. It is classified as “Generally Regarded as Safe” by both the European Food Safety Administration (EFSA) and US Food and Drug Administration (FDA).

The Acetium capsule used in this study is a CE-marked product and the formulation is registered in many countries as a medical device (class IIa). This classification is based on the nature of the active ingredient, L-cysteine, as a natural amino acid and the mode of the action of the formulation. Characteristically, slow-release L-cysteine locally acts in the stomach, where acetaldehyde is also formed. Acetium capsules include 100 mg of L-cysteine, which is bound to matrix granules with a matrix former. This causes L-cysteine to be released at a sustained rate and locally in the stomach [25]. The use of a multi-particle system ensures that the formulation spreads to as large a part of the stomach as possible, even when the stomach contains solid or semi-solid content (i.e. food). Granules can also be assumed to remain longer in gastric mucosal folds, even in an upright position. After its release from the granules, L-cysteine quickly dissolves in the stomach and reacts with acetaldehyde, forming inactive 2-methylthiazolidine-4-carboxylic acid [22].

It has earlier been demonstrated that slow-release L-cysteine capsules effectively eliminate ethanol-derived acetaldehyde in the gastric juice of patients with atrophic gastritis [25]. In that study, patients orally ingested a 15% alcohol solution in a total dose of 0.3 g/kg. The effect of L-cysteine capsules (4 x 50 mg) was documented to last for at least 40 minutes [25]. In the present study, slowly L-cysteine releasing capsules (2 x 100 mg) reduced gastric juice acetaldehyde levels by a mean of 67% and 60% in PPI-treated ALDH2-actives and -deficients, respectively. The acetaldehyde eliminating action of L-cysteine could thus be documented to be effective even after a moderate dose (0.5 g/kg) of alcohol and to last for 2 hours.

L-cysteine capsules had no effect on salivary acetaldehyde concentrations in either ALDH2-active or -deficient subjects. This finding underlines the localized effect of a slowly L-cysteine releasing capsule in the stomach.

### Preventive aspects

Stomach cancer is the third leading cause of cancer death in both sexes worldwide (723,000 deaths, 8.8% of the total) [36]. The highest estimated mortality rates are in Eastern Asia (24 per 100,000 in men, 9.8 per 100,000 in women). Upper aerodigestive tract cancers have a particularly poor prognosis. With regard to gastric cancer, the six-month survival rate is 65% in those diagnosed in the early stages and less than 15% in those diagnosed in the late stages, and the five-year survival rate is between 5–10%, making the prevention of stomach cancers extremely important [36].

Cancer prevention is based on the identification of specific etiological factors, e.g. ALDH2 deficiency, achlorhydric atrophic gastritis, *Helicobacter pylori* infection, and group 1 carcinogenic agents. ALDH2 deficiency appears to be the most prevalent and powerful known genetic risk factor for cancer. It has been calculated to affect at least 540 million individuals with Eastern Asian roots [4,37]. A mutant allele encoding an inactive subunit of aldehyde dehydrogenase-2 (*ALDH2*; rs671) was carried by Han Chinese as they spread throughout Eastern Asia, i.e. to the countries with the highest incidence of esophageal and gastric cancers [38]. ALDH2-deficient heavy drinkers have been shown to have an over 100-fold higher risk of esophageal cancer [37,39]. ALDH2 deficiency can be screened with a simple flushing questionnaire and/or ethanol path test [4,37]. It has been calculated that if moderate or heavy drinking ALDH2 heterozygotes were instead only light drinkers, 53% of esophageal squamous cell carcinomas might be prevented in the Japanese male population [4,37].

In this study, gastric juice ethanol levels from 60 min to 120 min time points were in general clearly lower than the limit (1.0 vol %) for Japanese official alcoholic beverages. Thus, especially in ALDH2 deficiency and/or achlorhydria, acetaldehyde accumulates in the gastric juice even at low ethanol concentrations (0.05–1%, i.e. 0.5–10‰ or 10–200 mM), representing the levels found in many foodstuffs and beverages produced by fermentation, e.g. pickled food, soya sauces and dairy products [40–42]. Due to the relatively slow gastric emptying rate and effective first pass metabolism of ethanol, these products do not normally lead to significant systemic effects of ethanol, but especially in ALDH2-deficent and/or achlorhydric subjects may result in prolonged exposure of the gastric mucosa to carcinogenic acetaldehyde.

A recent meta-analysis demonstrated that compared with nondrinkers, heavy drinking but not moderate drinking significantly associated with gastric cancer [14]. On the other hand, several other studies have reported increased incidences of gastric cancer among ALDH2-deficient nondrinkers and as well as among high consumers of foodstuffs produced by fermentation [43–47]. Our current results on gastric juice acetaldehyde production at low ethanol concentrations could explain the latter and so far unrecognized association. In addition, high concentration of carcinogenic acetaldehyde produced in the gastric juice of ALDH2-deficent and/or achlorhydric subjects could also be involved in the esophageal carcinogenesis through gastro-esophageal reflux of the gastric contents [48].

ALDH2 deficiency, achlorhydria and alcohol-associated exposure to intragastric acetaldehyde appear to play a key role in the pathogenesis of gastric cancer. Therefore, the screening and health education of specific risk groups, including those with ALDH2 deficiency, atrophic gastritis, *H. pylori* infection, and regular users of PPI drugs, could have a major impact on the prevention of not only oral and esophageal cancers, but also gastric cancer. The health education should include information on the acetaldehyde and ethanol concentrations of official alcoholic beverages, and as well as foodstuffs and beverages containing carcinogenic levels of acetaldehyde and/or low concentrations of alcohol.

It is generally agreed that the ALARA principle (“As Low As Reasonably Achievable”) should be applied to all mutagenic and carcinogenic agents. Slowly L-cysteine releasing formulations effectively eliminate carcinogenic acetaldehyde in the saliva and gastric juice. However, their actual effectiveness in cancer prevention remains to be evaluated in prospective intervention studies.

### Strengths and limitations

A major strength of the study is the highly significant and marked increase in the gastric juice and salivary acetaldehyde concentrations in every study (1, 2 and 3) after intragastric administration of alcohol. ALDH2 deficiency resulted in a 5.6-fold and PPI treatment in a 1.5- to 3.0-fold increase in the acetaldehyde exposure of the gastric mucosa to carcinogenic acetaldehyde.

Another strength of the study is that the effect of alcohol on gastric juice and salivary acetaldehyde could be demonstrated after a moderate dose of alcohol. With this dose, the increasing effect of ALDH2 deficiency and PPI treatment and the decreasing effect of L-cysteine on gastric juice acetaldehyde could be demonstrated to last for almost the whole follow-up period of 120 minutes.

The major limitations of the study are as follows. At some time points it was impossible to aspirate gastric juice without water infusion before sampling. This diluted some of the samples and resulted in marked individual variations in some cases and time points. However, this limitation in fact strengthens our findings, since the real gastric juice acetaldehyde concentration in the vicinity of mucosal surfaces was presumably higher than observed in this study.

Secondly, this study included only *H*.*pylori*-negative subjects. In addition to atrophic gastritis, *H*.*pylori* infection is also a major risk factor for gastric cancer [49]. Many *H*.*pylori* strains possess the ADH enzyme and are able to produce carcinogenic acetaldehyde from ethanol [50]. Gastric juice acetaldehyde levels in the presence of ethanol thus remain to be examined in ALDH2-actives and -deficients with *H*.*pylori* infection.

Thirdly, tobacco smoking is an independent risk factor for stomach cancer [51]. Acetaldehyde is a major carcinogen of tobacco smoke, which readily dissolves in the saliva during smoking and has a synergistic effect via saliva with alcohol on the exposure of the upper aerodigestive tract to acetaldehyde [52]. The combined effect of tobacco smoking and alcohol ingestion on gastric juice acetaldehyde levels of ALDH2-actives and -deficients remains to be established.

### Conclusions

The alcohol-induced marked increase in gastric juice acetaldehyde in ALDH2-deficient subjects provides strong evidence for the local carcinogenic potential of acetaldehyde in gastric carcinogenesis. Nondependent changes in gastric juice and salivary acetaldehyde levels caused by ALDH2 deficiency, PPI treatment, and intragastric L-cysteine indicate that the gastric juice acetaldehyde concentration is locally regulated by gastric mucosal ADH- and ALDH2-enzymes and by oral microbes colonizing the acid-free (achlorhydric) stomach. Gastric juice acetaldehyde levels are highest when the intragastric ethanol concentrations are high, underlining the important role of alcoholic beverages in local acetaldehyde exposure. However, significant elevations of gastric juice acetaldehyde levels were also found at rather low ethanol concentrations, corresponding to those of many foodstuffs, beverages, and dairy products produced by fermentation. All this information provides entirely novel perspectives for the prevention of gastric cancer, especially among particular risk groups: those with ALDH2 deficiency, atrophic gastritis, *Helicobacter pylori* infection, and regular use of proton pump inhibitors.
